# Predicting microvascular invasion in solitary hepatocellular carcinoma: a multi-center study integrating clinical, MRI assessments, and radiomics indicators

**DOI:** 10.3389/fonc.2025.1511260

**Published:** 2025-02-19

**Authors:** Wang Jian, Lin Zhan, Lin Zhaowang, Yang Ling, Yu Min, Xie Rong, Lin Wanxing, Yang Yongfei, Tu Haibin

**Affiliations:** ^1^ Department of Radiology, Mengchao Hepatobiliary Hospital of Fujian Medical University, Fuzhou, China; ^2^ Biobank, Mengchao Hepatobiliary Hospital of Fujian Medical University, Fuzhou, China; ^3^ The United Innovation of Mengchao Hepatobiliary Technology Key Laboratory of Fujian, Fuzhou, China; ^4^ Department of Radiology, Datian County General Hospital, Sanming, China; ^5^ Department of Radiology, Second Hospital of Nanping City, Nanping, China; ^6^ Department of Ultrasound, Mengchao Hepatobiliary Hospital of Fujian Medical University, Fuzhou, China

**Keywords:** microvascular invasion (MVI), radiomics, MRI, multi-center study, predictive modeling

## Abstract

**Background:**

Microvascular invasion (MVI) is a key prognostic factor in solitary hepatocellular carcinoma (HCC), significantly affecting treatment decisions and outcomes. Early prediction of MVI is crucial for enhancing clinical decision-making.

**Objectives:**

This study aimed to develop and evaluate four predictive models for MVI: one based on clinical indicators, one on MRI assessments, one using radiomics, and a combined model integrating all data across multiple medical centers.

**Methods:**

The study included patients with solitary HCC from three centers (Mengchao Hepatobiliary Hospital, The Second Hospital of Nanping, and Datian County General Hospital). The dataset was divided into an internal training set, validation set, and two external validation sets. Predictive models were built using clinical indicators, MRI, radiomics, and a combination of these. Model performance was assessed through ROC curves, calibration curves, and decision curve analysis (DCA). Lasso regression identified significant features, and SHAP analysis interpreted the model predictions.

**Results:**

A total of 319 patients were analyzed: 199 from the internal center, 67 from Nanping, and 53 from Datian. The combined model, which integrated clinical, MRI, and radiomics features, showed superior performance, with an AUC of 0.95(95%CI:0.92-0.98) in the internal training set, 0.92(95%CI:0.83-1.00) in the internal validation set, 0.96(95%CI:0.92-1.00) in Nanping, and 0.94(95%CI:0.88-0.99) in Datian. Calibration curves confirmed the model’s accuracy, and NRI/IDI analyses highlighted its advantage over individual models. Key predictive indicators included pseudocapsule, peritumoral enhancement, and wavelet-based MRI features.

**Conclusion:**

This multi-center study demonstrates the effectiveness of combining clinical, MRI, and radiomics data in predicting MVI in solitary HCC, with robust results across different medical centers. These models have potential to improve patient management and treatment planning.

## 1 Introduction

Hepatocellular carcinoma (HCC) remains the most common form of primary liver cancer and a major contributor to cancer-related mortality globally (1). Its incidence continues to rise, particularly in regions with high prevalence of hepatitis B and C viruses, as well as among patients with chronic liver disease and cirrhosis (2, 3). Among the many prognostic factors linked to HCC, MVI has emerged as a crucial determinant of patient outcomes (4, 5). MVI is defined as the presence of tumor cells within the small vessels of the liver surrounding the tumor (6, 7), and its presence is associated with higher recurrence rates, metastasis, and decreased overall survival (8, 9).

Traditional methods for predicting MVI rely heavily on histopathological analysis and conventional MRI assessments modalities such as ultrasound and computed tomography (CT) (10). While these techniques are essential in clinical practice, they are limited by subjectivity, inter-observer variability, and their inability to fully capture the heterogeneity of HCC (11). Furthermore, the majority of these methods are dependent on post-operative pathology (12), restricting their utility in pre-surgical decision-making. As a result, there is an urgent need for non-invasive, accurate, and reliable methods to predict MVI preoperatively (13).

Recent advances in radiomics have shown great promise in extracting high-dimensional features from MRI assessments data, offering an opportunity to enhance the predictive accuracy of MVI assessments (14). Despite these advancements, most existing studies have focused on single-center datasets and individual data modalities, often ignoring the potential of integrating clinical, MRI assessments, and radiomics features (15). Such integration could provide a more holistic view of the tumor’s biological behavior and improve predictive performance. Moreover, many current machine learning and deep learning models, particularly those applied to radiomics, lack interpretability, which hinders their adoption in clinical settings (16).

In this multi-center study, we aim to address these gaps by integrating data from multiple centers to build a more robust and generalizable predictive model for MVI in solitary HCC. This study utilizes clinical, MRI assessments, and radiomics data from three medical centers, allowing us to assess the consistency and accuracy of our predictive models across diverse populations. Additionally, we employ a Transformer model to process the high-dimensional radiomics data, as this model excels at capturing complex relationships within large datasets. To improve model interpretability, we utilize SHAP (SHapley Additive exPlanations) analysis, which allows for a detailed examination of how individual features contribute to the model’s predictions.

By developing and validating a combined model that incorporates clinical, MRI assessments, and radiomics data across multiple centers, this study seeks to establish a comprehensive and reliable framework for predicting MVI in solitary HCC. The findings from this multi-center study have the potential to improve clinical decision-making, personalize treatment strategies, and enhance patient outcomes across different healthcare settings.

## 2 Materials and methods

### 2.1 Study design and population

**Figure 1:**
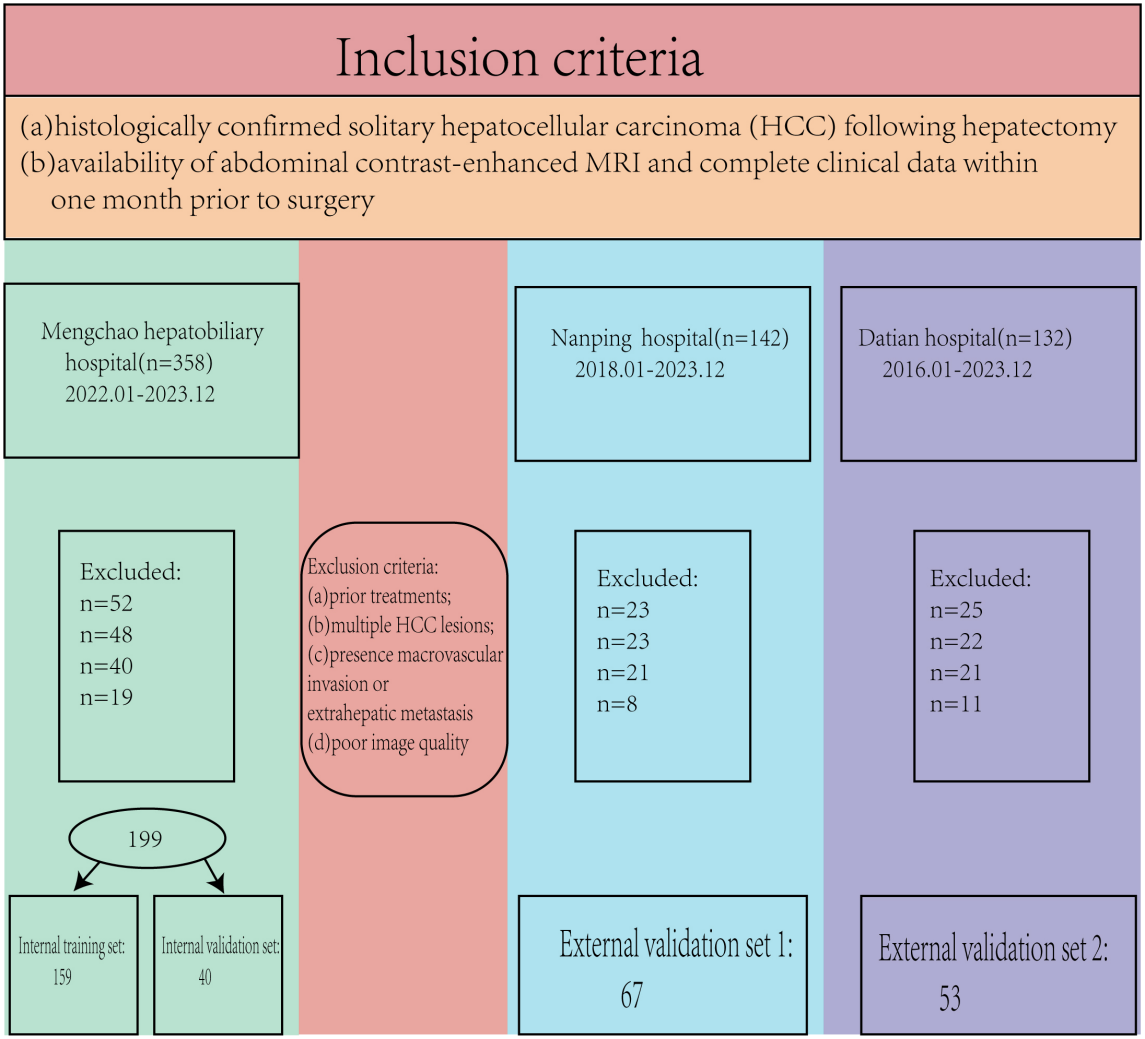
The patient inclusion flowchart.

This retrospective study was approved by the hospital’s ethics committee (No. 2020-010-01), and the requirement for written informed consent was waived as no patient-identifiable information was used. The study was conducted in accordance with the ethical principles outlined in the Declaration of Helsinki. A total of 632 patients (2018.01-2023.12) met the initial inclusion criteria; however, 313 patients were excluded based on the following criteria. The inclusion criteria were: (a) histologically confirmed solitary hepatocellular carcinoma (HCC) following hepatectomy, and (b) availability of abdominal contrast-enhanced MRI and complete clinical data within one month prior to surgery. Exclusion criteria were: (a) prior treatments for HCC before hepatectomy, (b) multiple HCC lesions, (c) presence of macrovascular invasion or extrahepatic metastasis, and (d) poor image quality (**Figure 1**).

### 2.2 Serological biomarker collection

Comprehensive serological data were retrospectively collected for all patients prior to surgery. Within one week before the surgery, the patient fasted for more than eight hours, and blood samples were collected. The biomarkers included key indicators of liver function, coagulation status, and tumor markers: alanine aminotransferase (ALT), aspartate aminotransferase (AST), total bilirubin (TBIL), direct bilirubin (DBIL), indirect bilirubin (IBIL), alkaline phosphatase (ALP), gamma-glutamyl transferase (GGT), prothrombin time (PT), activated partial thromboplastin time (APTT), international normalized ratio (INR), albumin (ALB), alpha-fetoprotein (AFP), carcinoembryonic antigen (CEA), carbohydrate antigen 19-9 (CA199), and carbohydrate antigen 125 (CA125). These biomarkers were measured using standardized laboratory assays as part of the preoperative assessment to evaluate liver function, coagulation status, and tumor burden. The collected data were then integrated with imaging and pathological findings for further analysis.

### 2.3 Pathological diagnosis of MVI

The diagnosis of MVI was determined through histopathological examination of resected tumor specimens using the seven-point sampling method as a reference. MVI was defined as the presence of cancer cells within the microvasculature, including small vessels in the tumor capsule or the surrounding liver tissue, as observed under a microscope. The assessment was performed independently by two experienced pathologists at each center. In cases where discrepancies in the diagnosis occurred, a third senior pathologist was consulted, and the final diagnosis was reached through consensus. This process ensured the accuracy and reliability of MVI detection across all cases.

### 2.4 MRI assessments data

#### 2.4.1 MRI evaluation

MRI assessments were performed using scanners with varying field strengths (Mengchao: Simens Verio 3.0T. Nanping: Philips Achieva 1.5T. Datian: GE Mr355 1.5T) across the participating centers. Sequences included T1-weighted images (pre-contrast, arterial, portal venous, and delayed phases) and T2-weighted images. The imaging protocols were standardized across centers, with the following parameters: slice thickness of 5 mm, inter-slice gap of 1 mm, field of view (FOV) of 380 mm × 380 mm, and matrix size of 512 × 512. These parameters provided detailed visualization of the tumor’s characteristics and vascularity. Two experienced radiologists independently reviewed the images and recorded the following parameters: tumor diameter, MRI LI-RADS classification, presence of intratumoral necrosis, pseudocapsule formation, liver cirrhosis, smoothness of the tumor margin, and peritumoral enhancement (17). Any discrepancies in the assessment were resolved through discussion to ensure consistency in data collection.

#### 2.4.2 Radiomics feature extraction

Radiomics features were extracted using 3D Slicer software (version 4.1.1). Two senior radiologists independently delineated the regions of interest (ROIs) for each tumor. While 3D Slicer provides a robust platform for radiomics feature extraction, it has certain limitations, such as its reliance on manual delineation, which introduces variability, and the lack of native support for some advanced preprocessing techniques. These limitations were mitigated in our study through stringent quality control, inter- and intra-observer agreement assessments, and feature standardization. To evaluate the reliability of the extracted features, the inter- and intra-observer agreement was quantitatively assessed using intraclass correlation coefficients (ICCs).

#### 2.4.3 Inter-observer agreement

The ICCs for radiomics features extracted by the two radiologists were calculated. Features with ICC values greater than 0.75, indicating excellent agreement, were retained for further analysis.

#### 2.4.4 Intra-observer agreement

To assess intra-observer consistency, one radiologist repeated the ROI delineation and feature extraction one month after the initial analysis. The ICCs between the two rounds of feature extraction were calculated, and only features with high intra-observer agreement (ICC > 0.75) were included in the subsequent analyses.

By ensuring that only features demonstrating high inter- and intra-observer agreement were retained, this rigorous process enhanced the reliability and reproducibility of the radiomics data used in the study.

#### 2.4.5 Data preprocessing

Prior to model construction, radiomics features underwent normalization to ensure comparability across different scales. Z-score normalization was applied to standardize the features by centering them around a mean of 0 and scaling to a standard deviation of 1. Initial feature selection was conducted using variance thresholds to eliminate low-variance features, ensuring that only informative features were retained. Variance threshold filtering was chosen as it is a straightforward and efficient method for removing features with minimal information. Compared to alternative methods like principal component analysis (PCA) or recursive feature elimination (RFE), this approach balances computational efficiency and interpretability, making it particularly suitable for clinical data. Subsequent LASSO regression was used to refine the feature set further. Additionally, correlation analysis was performed to identify and remove highly correlated features, thereby reducing redundancy.

### 2.5 Model construction

#### 2.5.1 Radiomics model

A Transformer model was first constructed to process the high-dimensional radiomics features. This model architecture included multiple self-attention layers, allowing it to effectively capture complex relationships within the data. The model was trained using a dataset split into training and validation groups, optimizing parameters such as learning rate, batch size, and the number of epochs through grid search methods.

#### 2.5.2 Feature selection using Lasso

To enhance model interpretability and identify the most significant predictors of MVI, Lasso regression was applied post-training. This technique performed regularization, effectively shrinking coefficients of less important features to zero, thereby facilitating dimensionality reduction. The resulting significant features were analyzed to identify key indicators associated with MVI, which provided insights into the underlying biological processes.

#### 2.5.3 Clinical indicator model

In addition to the radiomics model, a clinical indicator model was developed using Lasso regression for feature selection based on serum biomarkers and clinical data. Significant predictors identified through this method were integrated into a logistic regression model to assess their predictive capability.

#### 2.5.4 MRI assessment model

For the MRI assessment model, relevant indicators such as tumor size, edge characteristics, and LI-RADS classification were extracted and evaluated. LASSO regression was employed to construct this model. LASSO was chosen because of its ability to perform variable selection and regularization simultaneously, ensuring that the most relevant predictors were retained while reducing the risk of overfitting.

#### 2.5.5 Combined model construction

To construct a comprehensive predictive model, we employed a Transformer-based approach to integrate clinical, imaging, and radiomics features. The Transformer architecture included multiple self-attention layers with a multi-head attention mechanism and feed-forward neural networks. The Transformer model was specifically chosen for its ability to capture complex, non-linear interactions between features, making it highly suited for integrating high-dimensional, heterogeneous datasets. Compared to traditional machine learning models, such as logistic regression or random forests, the Transformer excels at modeling dependencies between diverse modalities. Unlike convolutional neural networks (CNNs), which are effective for spatial data, the Transformer is more versatile in handling non-image data types like clinical and radiomics features. This versatility was crucial for our study, as it involved combining imaging and non-imaging data into a unified predictive framework. Positional encoding was incorporated to retain feature order. Inputs were normalized and converted into a unified feature matrix, treating each feature as an independent token. The model was trained using Adam optimizer (learning rate = 0.001) with binary cross-entropy loss. Dropout (rate = 0.2) was used for regularization. Hyperparameters, such as the number of attention heads, Transformer layers, learning rate, dropout rate, and batch size, were optimized via grid search. The area under the receiver operating characteristic curve (AUC) on the validation set was used as the primary metric for evaluation. The final model used 8 attention heads, 4 Transformer layers, a learning rate of 0.001, a dropout rate of 0.2, and a batch size of 32, which provided the highest AUC on the validation set. The output layer provided binary classification, and SHAP analysis was used for interpretability, enabling the model to capture complex interactions among the diverse input features. Key clinical biomarkers (e.g., ALT, AST, AFP, ALB), MRI features (e.g., tumor size, LI-RADS classification, necrosis, pseudocapsule, liver cirrhosis, tumor margin smoothness, peritumoral enhancement), and radiomics features were combined to form a unified feature set.

### 2.6 SHAP Analysis

Following model construction, SHAP analysis was performed to interpret the contributions of individual features to model predictions. SHAP values were calculated for each feature, with visualizations including waterfall plots, swarm plots, and force plots created to illustrate feature importance and their impact on MVI predictions (18).

### 2.7 Statistical analysis

The continuous data are presented as mean ± standard deviation, and categorical data are presented as percentages. When variance homogeneity and normal distribution are satisfied, continuous data are analyzed using multivariate analysis or independent two-sample t-tests. Otherwise, the Kruskal-Wallis test or Mann-Whitney U test is used. Categorical data are analyzed using the chi-square test. The predictive efficiency between different models is assessed by plotting ROC curves, calculating the AUC and the corresponding 95% confidence intervals for each model, and using the Delong test for AUC comparison. Sensitivity, specificity, positive predictive value, negative predictive value, accuracy, and the corresponding 95% confidence intervals for each model are calculated. Calibration curves for each model are plotted, and the corresponding Hosmer-Lemeshow test and Brier score are calculated. Clinical decision curves (DCA) for each model are drawn, and the corresponding Net Reclassification Improvement (NRI) and Integrated Discrimination Improvement (IDI) are calculated. All comparisons are conducted using two-sided tests, with P < 0.05 considered statistically significant. Radiomics features were extracted using 3D Slicer software (version 4.11.20210226). Statistical analyses were conducted using R (version 4.1.1) and Python (version 3.6.5), with relevant packages including Scikit-learn (version 0.24.2) for machine learning, SHAP (version 0.39.0) for interpretability, and Matplotlib (version 3.3.4) for visualization.

## 3 Results

### 3.1 Patient characteristics

A total of 319 patients were included in this multi-center study, with 199 patients from the internal center and 120 patients from two external centers (67 from Nanping Hospital and 53 from Datian Hospital). Among the total cohort, 267 (83.7%) were male, and 52 (16.3%) were female, with a mean age of 57.4 ± 11.1 years. There were no significant differences in age across the internal and external cohorts (p = 0.83) (**Table 1**).

**Table 1:** Basic characteristics in all patients.

	Total	Internal Training	Internal Testing	Nanping hospital	Datian hospital	P-value
MVI						
non-MVI	153 (48.0%)	78 (49.1%)	17 (42.5%)	29 (43.3%)	29 (54.7%)	0.55
MVI	166 (52.0%)	81 (50.9%)	23 (57.5%)	38 (56.7%)	24 (45.3%)	
**Diameter(cm)**	3.4 ± 2.0	3.3 ± 2.0	3.3 ± 1.9	3.5 ± 2.0	3.5 ± 1.9	0.88
**Age(years)**	57.4 ± 11.1	57.1 ± 10.7	57.6 ± 13.7	58.5 ± 11.8	56.9 ± 9.7	0.83
**ALT(U/L)**	36.0 ± 25.9	34.2 ± 21.2	44.5 ± 44.3	35.5 ± 24.2	35.5 ± 21.5	0.16
**AST(U/L)**	36.2 ± 28.7	35.5 ± 25.5	39.1 ± 42.2	34.5 ± 29.6	38.2 ± 24.1	0.8
**TBIL(umol/L)**	14.9 ± 7.8	14.9 ± 7.9	13.2 ± 5.8	15.3 ± 9.1	15.3 ± 7.2	0.55
**DBIL(umol/L)**	5.3 ± 3.2	5.5 ± 3.2	5.0 ± 2.8	5.7 ± 2.9	4.6 ± 4.0	0.2
**IBIL(umol/L)**	9.5 ± 5.3	9.4 ± 5.4	8.2 ± 3.7	9.6 ± 6.4	10.7 ± 4.4	0.17
**ALP(U/L)**	89.7 ± 38.8	88.4 ± 33.3	93.5 ± 62.8	84.9 ± 22.3	96.7 ± 46.6	0.35
**GGT(U/L)**	65.3 ± 95.3	68.0 ± 114.2	59.4 ± 78.5	61.1 ± 63.3	67.0 ± 78.2	0.94
**PT(s)**	13.4 ± 1.1	13.5 ± 1.1	13.3 ± 0.8	13.0 ± 0.6	13.8 ± 1.4	< 0.001
**APTT(s)**	35.7 ± 3.0	35.8 ± 3.1	35.8 ± 3.5	35.2 ± 3.5	36.3 ± 1.6	0.34
**INR**	1.0 ± 0.1	1.0 ± 0.1	1.0 ± 0.1	1.0 ± 0.1	1.1 ± 0.1	< 0.001
**ALB(g/L)**	42.8 ± 4.3	42.1 ± 3.9	43.4 ± 4.3	46.4 ± 2.5	39.9 ± 4.2	< 0.001
**AFP(ug/L)**	1107.8 ± 6022.3	1289.4 ± 6494.5	176.7 ± 519.1	1151.8 ± 7336.0	1209.7 ± 4934.2	0.77
**CEA(ng/ml)**	2.8 ± 2.0	2.8 ± 2.1	2.3 ± 1.2	3.0 ± 2.5	2.9 ± 1.7	0.43
**CA199(U/ml)**	19.6 ± 18.2	20.2 ± 18.0	17.8 ± 19.4	17.3 ± 15.5	22.4 ± 20.6	0.42
**CA125(U/ml)**	23.3 ± 90.9	23.2 ± 91.1	15.1 ± 16.4	12.4 ± 8.1	43.5 ± 156.1	0.27
LIRADS						
4	30 (9.4%)	16 (10.1%)	2 (5.0%)	7 (10.4%)	5 (9.4%)	0.83
5	289 (90.6%)	143 (89.9%)	38 (95.0%)	60 (89.6%)	48 (90.6%)	
Sex						
Female	52 (16.3%)	23 (14.5%)	8 (20.0%)	12 (17.9%)	9 (17.0%)	0.79
Male	267 (83.7%)	136 (85.5%)	32 (80.0%)	55 (82.1%)	44 (83.0%)	
Margin						
smooth	301 (94.4%)	151 (95.0%)	37 (92.5%)	65 (97.0%)	48 (90.6%)	0.43
coarse	18 (5.6%)	8 (5.0%)	3 (7.5%)	2 (3.0%)	5 (9.4%)	
Tumor necrosis						
no	302 (94.7%)	150 (94.3%)	39 (97.5%)	64 (95.5%)	49 (92.5%)	0.81
yes	17 (5.3%)	9 (5.7%)	1 (2.5%)	3 (4.5%)	4 (7.5%)	
Pseudocapsule						
no	96 (30.1%)	49 (30.8%)	11 (27.5%)	21 (31.3%)	15 (28.3%)	0.97
yes	223 (69.9%)	110 (69.2%)	29 (72.5%)	46 (68.7%)	38 (71.7%)	
Peritumoral enhancement						
no	174 (54.5%)	87 (54.7%)	21 (52.5%)	29 (43.3%)	37 (69.8%)	0.036
yes	145 (45.5%)	72 (45.3%)	19 (47.5%)	38 (56.7%)	16 (30.2%)	
Cirrhosis						
no	159 (49.8%)	68 (42.8%)	27 (67.5%)	35 (52.2%)	29 (54.7%)	0.031
yes	160 (50.2%)	91 (57.2%)	13 (32.5%)	32 (47.8%)	24 (45.3%)	
Family history						
no	260 (81.5%)	129 (81.1%)	32 (80.0%)	59 (88.1%)	40 (75.5%)	0.33
yes	59 (18.5%)	30 (18.9%)	8 (20.0%)	8 (11.9%)	13 (24.5%)	

ALT, Alanine Aminotransferase; AST, Aspartate Aminotransferase; TBIL, Total Bilirubin; DBIL, Direct Bilirubin; IBIL, Indirect Bilirubin; ALP, Alkaline Phosphatase; GGT, Gamma-Glutamyl Transferase; PT, Prothrombin Time; APTT, Activated Partial Thromboplastin Time; INR, International Normalized Ratio; ALB, Albumin; AFP, Alpha-Fetoprotein; CEA, Carcinoembryonic Antigen; CA199, Carbohydrate Antigen 19-9; CA125, Carbohydrate Antigen 125; LI-RADS, Liver Imaging Reporting and Data System.

Patients were divided into four groups: internal training set (n = 159), internal validation set (n = 40), Nanping hospital (n = 67), and Datian hospital (n = 53). The incidence of MVI was 52.0% (n = 166), with no statistically significant differences in MVI status between the internal and external groups (p = 0.55). Tumor characteristics, including diameter, liver function markers (ALT, AST), and other clinical parameters, were similar across groups (**Table 1**).

### 3.2 MVI and clinical features

Patients were further categorized by MVI status. In the internal training set, the MVI-positive group had a significantly larger tumor diameter (4.0 ± 2.2 cm) compared to the non-MVI group (2.6 ± 1.6 cm, p < 0.001) (**Supplementary Table 1**). The presence of cirrhosis was more common in the MVI-positive group (77.8%) compared to the non-MVI group (60.3%, p = 0.025) (**Supplementary Table 1**). Additionally, coarse tumor margins were observed more frequently in the MVI-positive group (75.3%) compared to the non-MVI group (14.1%, p < 0.001), indicating a strong association between margin characteristics and MVI (**Supplementary Table 1**). Other baseline characteristics, such as liver function tests (ALT, AST, TBIL, ALP), AFP levels, and the presence of a pseudocapsule, were not significantly different between the MVI and non-MVI groups (**Table 1**).

### 3.3 External validation

The external validation sets showed similar trends. In Nanping hospital, the MVI-positive rate was 56.7%, and in Datian hospital, it was 45.3%, with no significant differences in MVI incidence between the internal and external cohorts (p = 0.55) (**Table 1**). Tumor diameter, age, and other clinical features were consistent across the internal and external validation cohorts, demonstrating the robustness of the model across different centers.

### 3.4 ROC curves and AUC

**Figure 2:**
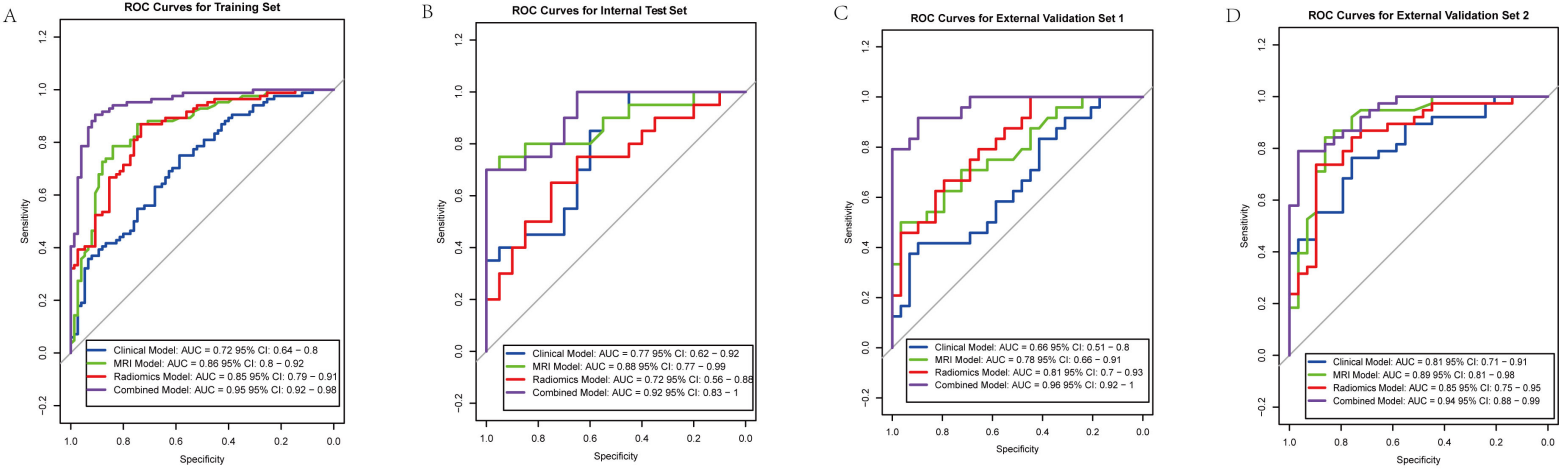
Receiver operating characteristic (ROC) curves for predictive models across different cohorts. **(A)** Training set: Combined model (AUC = 0.95), Clinical model (AUC = 0.72), MRI model (AUC = 0.86), Radiomics model (AUC = 0.85). **(B)** Internal validation set: Combined model (AUC = 0.92), Clinical model (AUC = 0.77), MRI model (AUC = 0.88), Radiomics model (AUC = 0.72). **(C)** External validation set 1: Combined model (AUC = 0.96), Clinical model (AUC = 0.66), MRI model (AUC = 0.78), Radiomics model (AUC = 0.81). **(D)** External validation set 2: Combined model (AUC = 0.94), Clinical model (AUC = 0.81), MRI model (AUC = 0.89), Radiomics model (AUC = 0.85).

The combined model outperformed the individual clinical, MRI, and radiomics models across both the internal and external validation sets (**Figure 2**). In the internal training set, the combined model achieved the highest AUC of 0.94, while in the internal validation set, it achieved an AUC of 0.90. In the external validation sets, the combined model continued to excel, with AUCs of 0.96 in Nanping hospital and 0.94 in Datian hospital.

Compared to the individual models, the combined model consistently showed superior predictive power, demonstrating the value of integrating clinical, MRI assessments, and radiomics features. Statistical comparisons using DeLong’s test confirmed that the AUC of the combined model was significantly higher than the clinical model (p < 0.001), MRI model (p = 0.002), and radiomics model (p = 0.003) in the internal training set. Similar statistically significant differences were observed across the internal test set and external validation datasets. Detailed performance metrics, including sensitivity, specificity, and accuracy, are presented in **Table 2**.

**Table 2:** Training and test set AUC/Hosmer-Lemeshow/Brier/Sensitivity/Specificity/PPV/NPV/accuracy.

		AUC	95%CI		Hosmer-Lemeshow	Brier score	Sensitivity (95%CI)	Specificity (95%CI)	Positive predict value (95%CI)	Negative predict value (95%CI)	Accuracy (95%CI)
			Lower	Upper							
Internal Training set	Clinical	0.73	0.66	0.81	0.814	0.212	0.75 (0.657, 0.843)	0.579 (0.468, 0.690)	0.663 (0.568, 0.758)	0.677 (0.563, 0.791)	0.669 (0.596, 0.742)
	MRI	0.85	0.79	0.91	0.244	0.148	0.786 (0.698, 0.873)	0.842 (0.760, 0.924)	0.846 (0.766, 0.926)	0.780 (0.691, 0.870)	0.813 (0.752, 0.873)
	Radiomics	0.83	0.76	0.89	0.604	0.159	0.821 (0.740, 0.903)	0.75 (0.653, 0.847)	0.784 (0.698, 0.870)	0.792 (0.698, 0.885)	0.788 (0.724, 0.851)
	Combined	0.94	0.91	0.97	0.516	0.095	0.893 (0.827, 0.959)	0.868 (0.792, 0.944)	0.882 (0.814, 0.951)	0.880 (0.806, 0.954)	0.881 (0.831, 0.931)
Internal Test set	Clinical	0.65	0.48	0.81	0.467	0.202	0.70 (0.499, 0.901)	0.684 (0.475, 0.893)	0.70 (0.499, 0.901)	0.684 (0.475, 0.893)	0.692 (0.547, 0.837)
	MRI	0.83	0.78	0.9	0.66	0.175	0.70 (0.499, 0.901)	0.947 (0.847, 1.0)	0.933 (0.807, 1.0)	0.75 (0.577, 0.923)	0.821 (0.700, 0.941)
	Radiomics	0.79	0.63	0.92	0.382	0.22	0.75 (0.560, 0.940)	0.684 (0.475, 0.893)	0.714 (0.521, 0.907)	0.722 (0.515, 0.929)	0.718 (0.577, 0.859)
	Combined	0.9	0.79	0.98	0.294	0.082	0.75 (0.560, 0.940)	0.842 (0.678, 1.0)	0.833 (0.661, 1.0)	0.762 (0.580, 0.944)	0.795 (0.668, 0.922)
Nanpin hospital	Clinical	0.66	0.51	0.8	0.531	0.251	0.700 (0.500, 0.850)	0.655 (0.520, 0.811)	0.710 (0.510,0.857)	0.655 (0.455,0.758)	0.642 (0.488,0.758)
	MRI	0.78	0.66	0.91	0.626	0.264	0.750 (0.620,0.902)	0.755 (0.645,0.864)	0.940 (0.814,1.00)	0.761 (0.588, 0.934)	0.802 (0.702,0.920)
	Radiomics	0.81	0.7	0.93	0.157	0.159	0.750 (0.620,0.902)	0.755 (0.645,0.864)	0.725 (0.511,0.857)	0.732 (0.521,0.934)	0.727 (0.547,0.869)
	Combined	0.96	0.92	1	0.248	0.062	0.805 (0.700,0.950)	0.775 (0.675,0.880)	0.855 (0.682,0.966)	0.768 (0.591,0.924)	0.800 (0.655, 0.950)
Datian hospital	Clinical	0.81	0.71	0.91	0.566	0.315	0.650 (0.485,0.800)	0.585 (0.478,0.700)	0.688 (0.500, 0.869)	0.668 (0.574,0.829)	0.584 (0.458,0.827)
	MRI	0.89	0.81	0.98	0.342	0.324	0.705 (0.520,0.866)	0.844 (0.750, 0.935)	0.824 (0.752,0.955)	0.752 (0.628,0.921)	0.811 (0.701,0.992)
	Radiomics	0.85	0.75	0.95	0.189	0.189	0.705 (0.520,0.866)	0.775 (0.662,0.858)	0.700 (0.515,0.868)	0.699 (0.578,0.842)	0.733 (0.604,0.900)
	Combined	0.94	0.88	0.99	0.375	0.057	0.805 (0.700,0.920)	0.869 (0.758, 0.954)	0.838 (0.627,0.995)	0.770 (0.613,0.922)	0.852 (0.714,0.988)

### 3.5 Calibration curves (Hosmer-Lemeshow test and Brier score)

**Figure 3:**
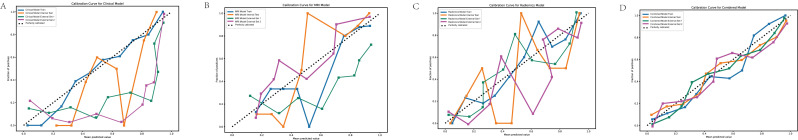
Calibration curves for predictive models. **(A)** Calibration curve for the clinical model. **(B)** Calibration curve for the MRI model. **(C)** Calibration curve for the radiomics model. **(D)** Calibration curve for the combined model. The calibration curves demonstrate the agreement between the predicted probabilities and observed outcomes for the four models. The combined model **(D)** shows the closest alignment with the ideal diagonal line across all datasets, indicating superior calibration compared to the clinical, MRI, and radiomics models.

The calibration curves for the combined model across all datasets, including both internal and external validation cohorts, demonstrated a strong alignment between predicted probabilities and actual outcomes (**Figure 3**). The Hosmer-Lemeshow test indicated good calibration with no significant deviations from a perfect fit. In the internal training set, the p-value was 0.516, and in the internal validation set, it was 0.294. In the external validation sets, the p-values were 0.248 for Nanping hospital and 0.375 for Datian hospital, further supporting the model’s well-calibrated nature. In terms of Brier score, the combined model consistently showed superior predictive accuracy across all cohorts. The Brier scores were lowest for the combined model compared to the individual clinical, MRI, and radiomics models, reflecting its superior probabilistic prediction accuracy. Specifically, the Brier score was 0.095 in the internal training set, 0.082 in the internal validation set, 0.062 in Nanping hospital set, and 0.057 in Datian hospital set (**Table 2**).

### 3.6 Decision curve analysis (DCA)

**Figure 4:**
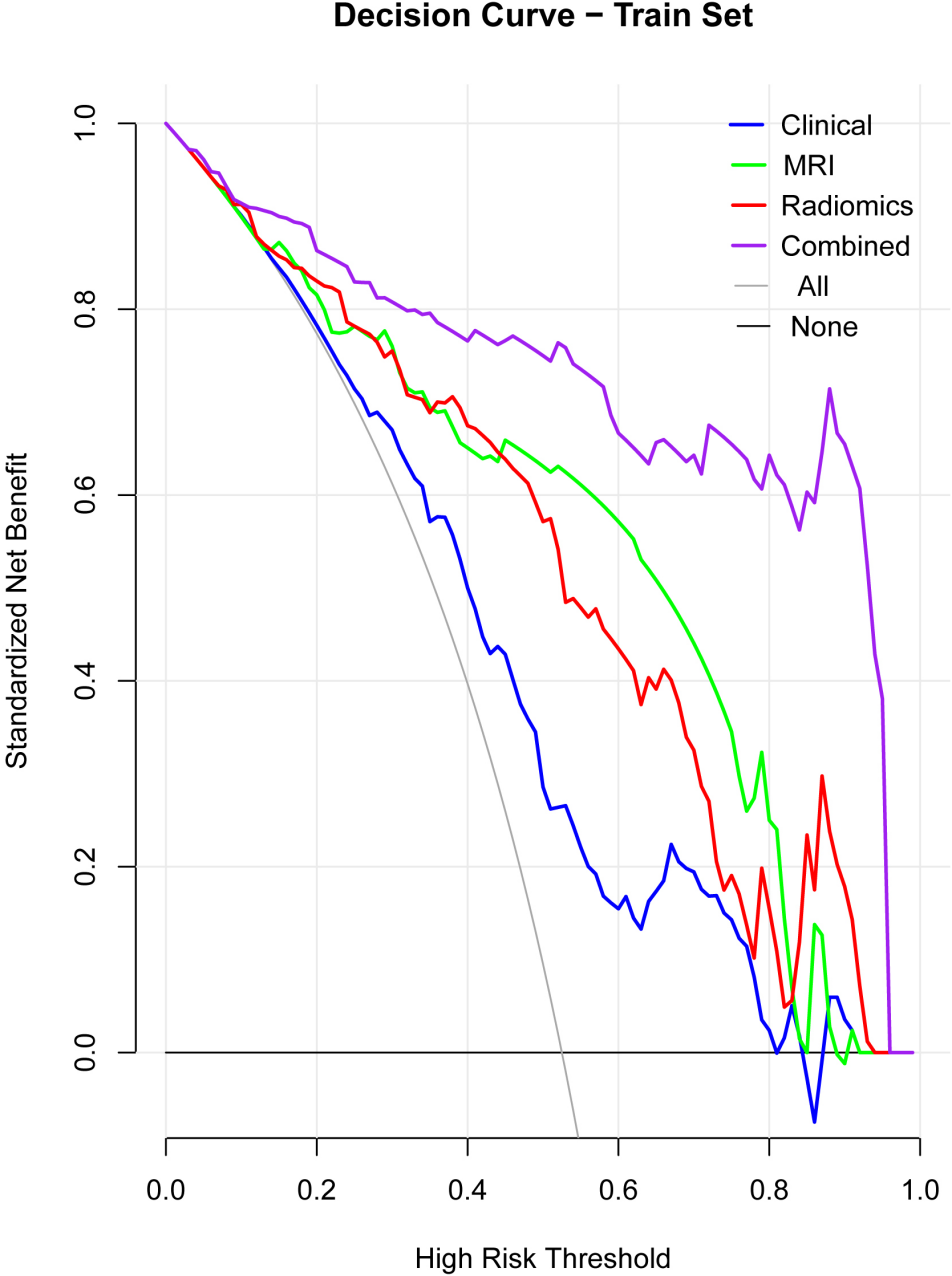
Decision curve analysis (DCA). The DCA curves illustrate the clinical net benefit of the clinical, MRI, radiomics, and combined models across a range of high-risk thresholds. The combined model consistently shows the highest net benefit across different thresholds, indicating superior clinical applicability compared to the individual models (clinical, MRI, radiomics). The “All” and “None” lines represent the extremes where all patients or no patients are assumed to be at risk.

DCA demonstrated that the combined model offered the greatest clinical benefit across a wide range of threshold probabilities compared to the individual models (**Figure 4**). This suggests the combined model’s predictions have better clinical applicability.

#### 3.6.1 NRI and IDI

Net Reclassification Improvement (NRI) and Integrated Discrimination Improvement (IDI) analyses showed that the combined model significantly outperformed the other models in reclassifying patients at risk of MVI (**Table 3**).

**Table 3:** NRI and IDI of combined model VS other models.

	NRI	Z value	P value	IDI	Z value	P value
Clinical	0.388	5.135	<0.001	0.412	5.747	<0.001
MRI	0.102	2.124	0.035	0.181	2.652	0.018
Radiomics	0.184	3.122	0.002	0.232	3.256	0.001

### 3.7 Lasso regression for feature selection

**Figure 5:**
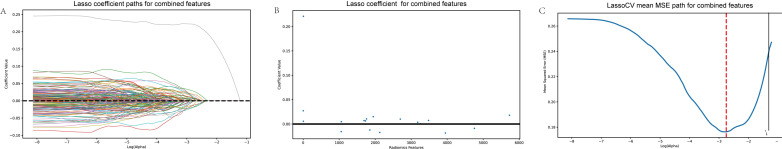
Lasso regression for feature selection. **(A)** Lasso coefficient paths for combined features. **(B)** Lasso coefficients after selection, indicating the most important features for predicting MVI. **(C)** Mean squared error (MSE) path from cross-validation (LassoCV), showing the optimal feature selection process. This figure illustrates the Lasso regression process, highlighting how important predictive features were identified and refined to enhance model performance.

Lasso regression was applied to identify the most important features in the combined model (**Figure 5**). The Lasso regression in clinical, MRI and radiomics were showed in **Supplementary Figures 1**–**3**. Key selected features included pseudocapsule, peritumoral enhancement, and wavelet-based radiomics indicators (For graphical aesthetics, abbreviate the names as follows:

‘pseudocapsule’: ‘PCapsule’,

‘Peritumoral_enhancement’: ‘PEnhancement’,

‘T1Arterywavelet_HHHglszmHighGrayLevelZoneEmphasis’: ‘T1A_HHH_GLZoneEmp’,

‘T1AArterywavelet_HHHglszmLowGrayLevelZoneEmphasis’: ‘T1A_HHH_LGZoneEmp’,

‘T1Portalwavelet_LHLglcmImc1’: ‘T1P_LHL_Imc1’,

‘T1Portalwavelet_LHLglszmGrayLevelNonUniformityNormalized’: ‘T1P_LHL_GLNonUnif’,

‘T1Portalwavelet_LHHfirstorderMedian’: ‘T1P_LHH_Median’,

‘T1Portalwavelet_HLLfirstorderMean’: ‘T1P_HLL_Mean’,

‘T1Portalwavelet_HLHfirstorderMedian’: ‘T1P_HLH_Median’,

‘T1Pwavelet_HHHfirstorderKurtosis’: ‘T1P_HHH_Kurtosis’,

‘T1Scan_sigma_1_5_mm_3DglszmSmallAreaLowGrayLevelEmphasis’: ‘T1S_SAL_GLZ’,

‘T1 Scan wavelet_HHLfirstorderMedian’: ‘T1S_HHL_Median’,

‘T1 Scan wavelet_HHLglszmSmallAreaEmphasis’: ‘T1S_HHL_SAEmphasis’,

‘T1delayoriginalfirstorderKurtosis’: ‘T1Delay_Kurtosis’,

‘T1delaywavelet_LHLfirstorderMedian’: ‘T1Delay_LHL_Median’,

‘T2log_sigma_0_5_mm_3DgldmDependenceVariance’: ‘T2Log_DepVariance’,

‘T2wavelet_LLLngtdmBusyness’: ‘T2Wavelet_Busyness’). The sort of importance showed in **Figure 6**.

**Figure 6:**
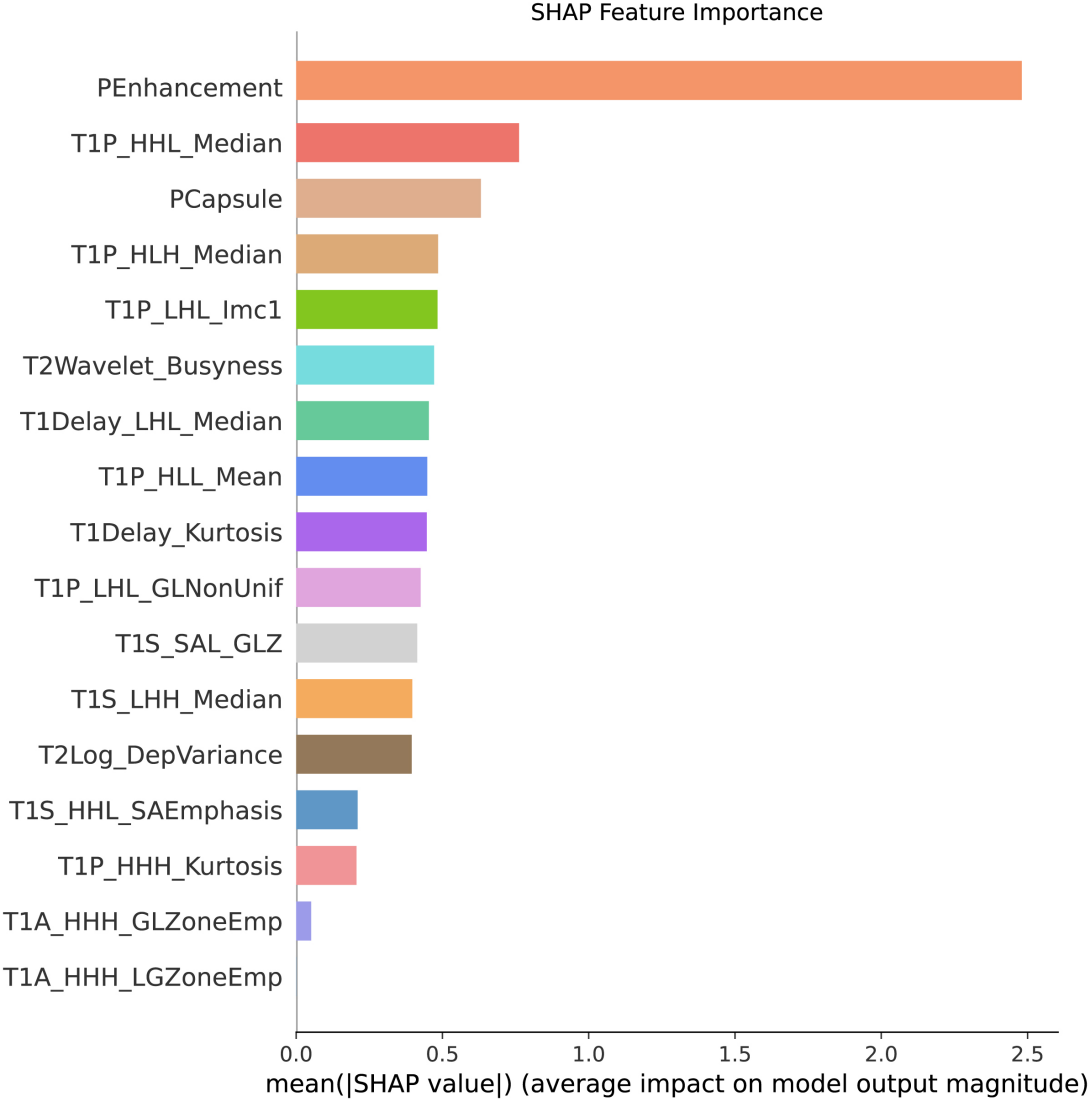
Feature importance ranking. This figure presents the sorted features based on their importance in predicting MVI. The features are ranked according to their contribution to the model’s predictive performance, with the most significant features, such as pseudocapsule and peritumoral enhancement, displayed at the top. These results highlight the key indicators driving the predictions in the combined model.

#### 3.7.1 Swarm plot

**Figure 7:**
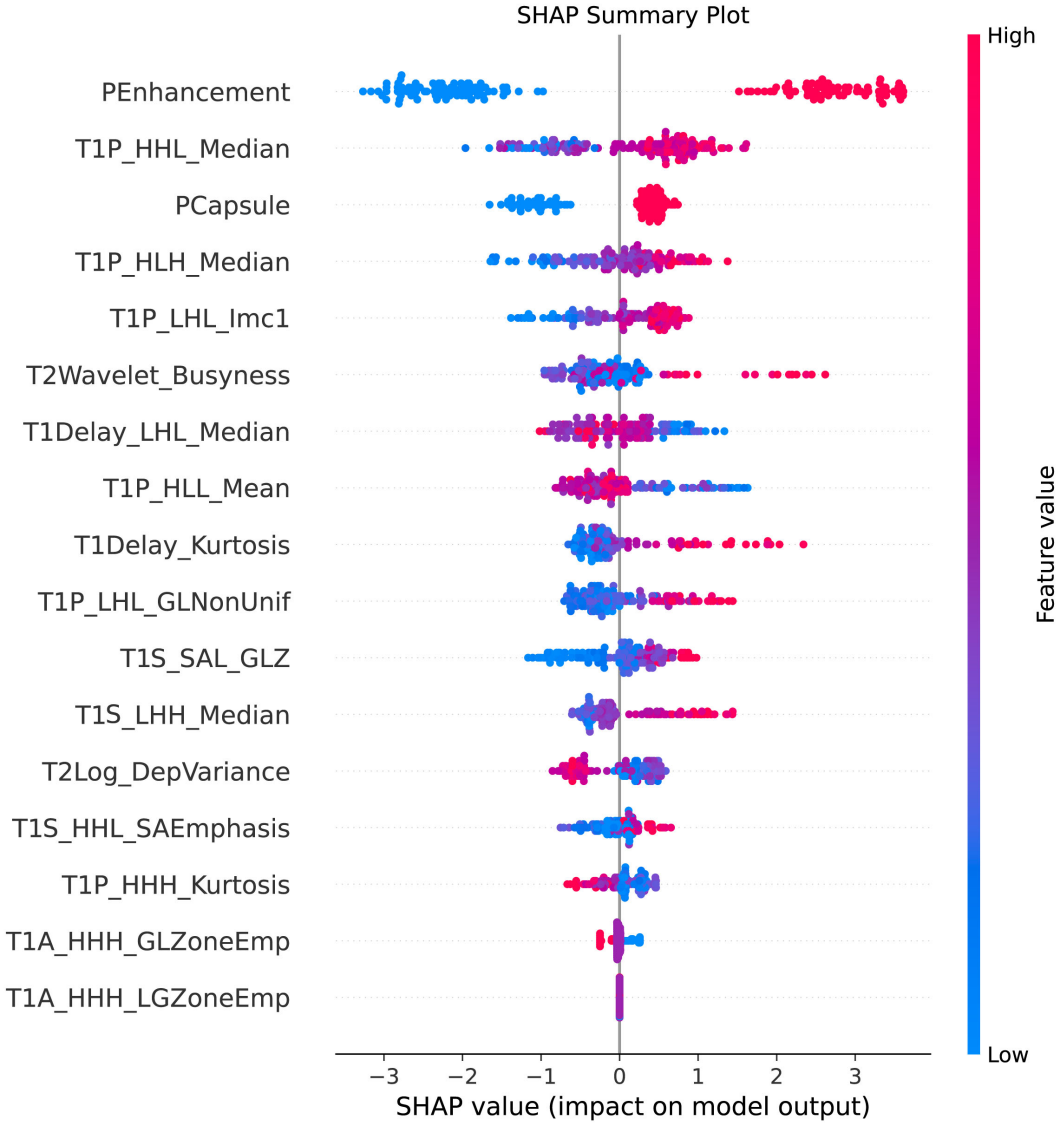
SHAP summary plot. This figure illustrates the SHAP (SHapley Additive exPlanations) summary plot, showcasing the impact of each feature on the model’s predictions. The features are ordered by their importance, with the color gradient indicating whether the feature value is high (red) or low (blue). The plot highlights how individual features influence the prediction of MVI, providing insights into the model’s decision-making process.

This plot illustrated the distribution of SHAP values for each feature, highlighting the direction and magnitude of their influence on the model’s predictions, showed in **Figure 7**.

#### 3.7.2 Force plot

**Figure 8:**
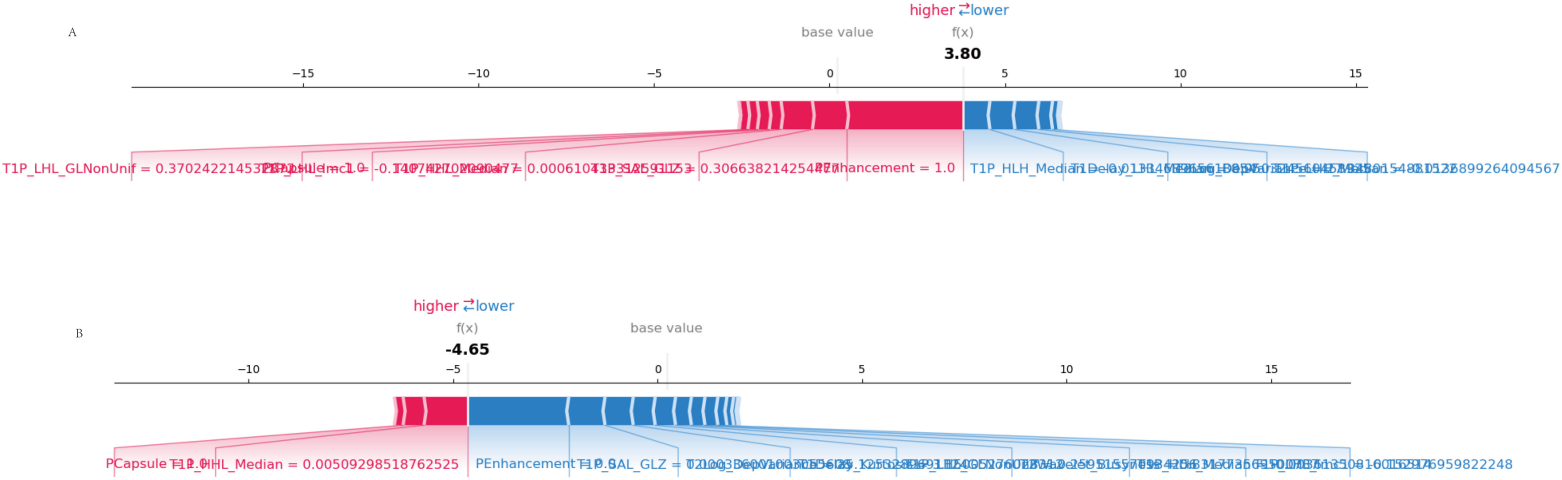
SHAP force plot. **(A)** Correct prediction: The SHAP force plot shows how individual features contribute to a correct prediction of MVI. Features in red push the prediction toward MVI, while features in blue push away from it. **(B)** Incorrect prediction: This SHAP force plot illustrates the contributions of individual features in a case where the model’s prediction of MVI was incorrect, highlighting the feature influences that led to the misclassification.

This visualization provided a clear representation of how each feature contributed positively or negatively to individual predictions for MVI (**Figure 8**).

#### 3.7.3 Waterfall plot

**Figure 9:**
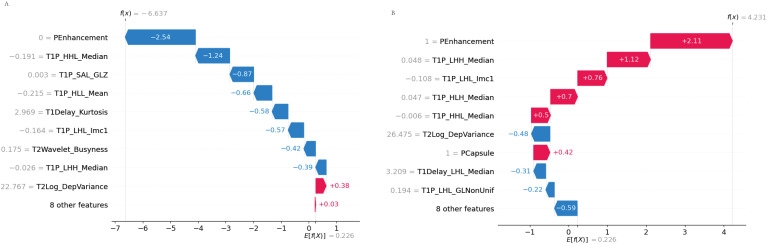
SHAP waterfall plot. **(A)** Accurate prediction: The waterfall plot illustrates how each feature contributes to the accurate prediction of MVI. Features pushing the prediction toward MVI are displayed in red, while those pushing it away are in blue. **(B)** Incorrect prediction: In this case, the waterfall plot shows how the combination of features led to an incorrect prediction, highlighting the cumulative influence of each feature in the misclassification.

The waterfall plot depicted the cumulative effect of the significant features on the predicted probabilities, allowing for an intuitive understanding of the model’s decision-making process (**Figure 9**).

#### 3.7.4 Dependence plot

**Figure 10:**
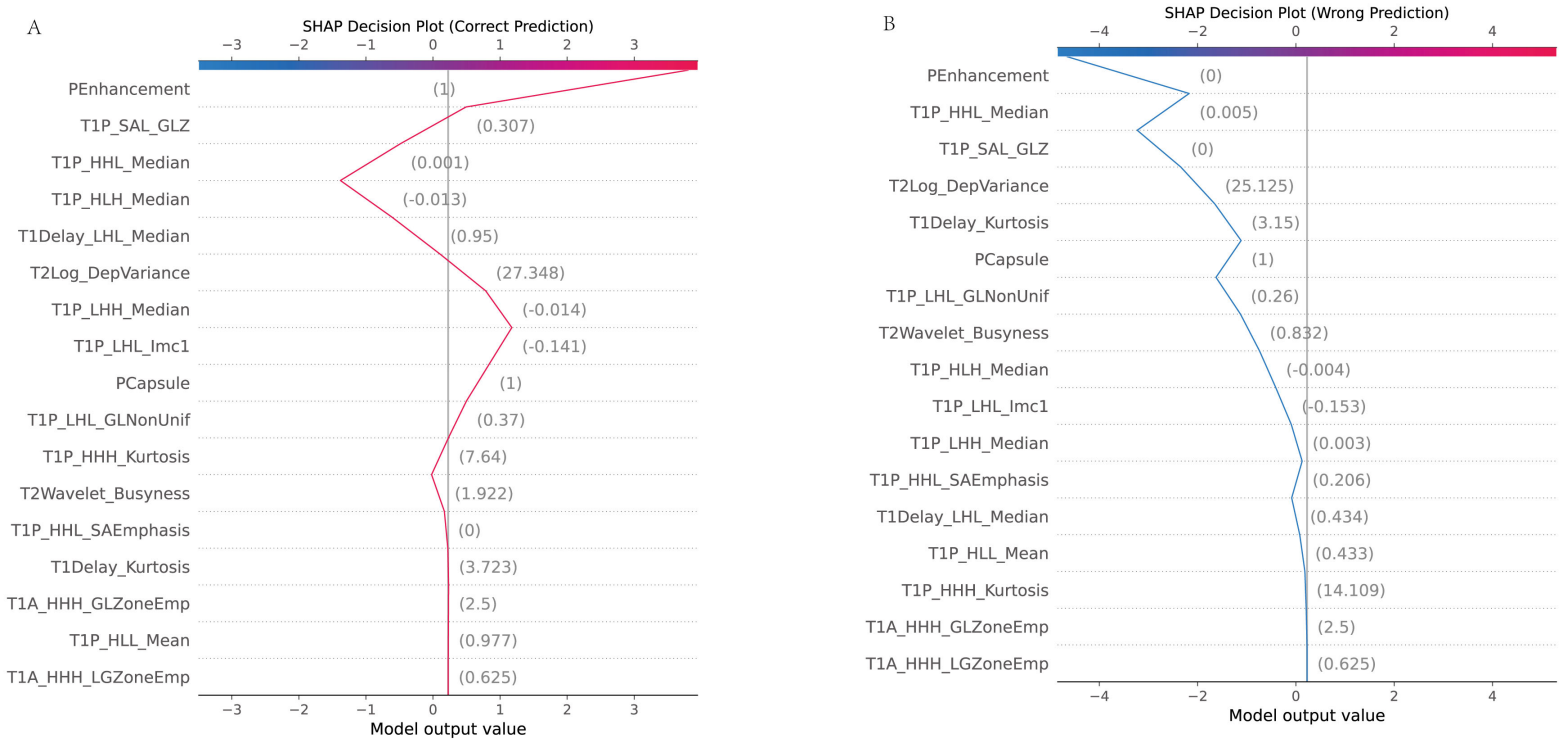
SHAP dependence plot. **(A)** Accurate prediction: The dependence plot shows how the value of a specific feature correlates with the SHAP value (the impact on the prediction) in a case where the model made a correct prediction. It demonstrates how changes in the feature’s value influence the prediction toward or away from MVI. **(B)** Incorrect prediction: This dependence plot illustrates a scenario where the feature’s value led to an incorrect prediction. It shows how this feature’s contribution may have misled the model, resulting in an inaccurate classification.

The dependence plot showcased the relationship between selected features and their SHAP values, indicating how variations in feature values influenced model predictions (**Figure 10**).

## 4 Discussion

In this multi-center study, we evaluated the predictive capability of a novel integrated model that combines clinical indicators, MRI assessments, and radiomics features across multiple medical centers to predict MVI in solitary hepatocellular carcinoma (HCC). Our results demonstrated that the combined model consistently outperformed individual models (clinical, MRI, and radiomics) in both internal and external validation sets, achieving the highest AUC across all datasets. This reinforces the value of integrating multimodal data in predicting MVI, aligning with previous studies that emphasize the enhanced predictive power of combining clinical and MRI assessments features. Notably, the multi-center design strengthens the generalizability of our findings.

One of the major strengths of our study is the inclusion of external validation cohorts, which helps mitigate the limitations often associated with single-center studies. The strong performance of the combined model in external validation sets highlights its robustness and potential applicability in diverse clinical settings. Importantly, features such as pseudocapsule and peritumoral enhancement consistently emerged as significant predictors of MVI, supporting their established roles in tumor invasiveness and vascular proliferation, as indicated in prior research (19, 20).

The clinical implications of our findings are significant, particularly in improving preoperative assessment and treatment planning for HCC patients. By accurately predicting MVI, our model could guide personalized treatment strategies, including decisions regarding surgical resection and adjuvant therapies, ultimately improving patient outcomes. Furthermore, the integration of advanced radiomics into standard diagnostic protocols could enhance the precision of preoperative risk stratification.

Mechanistically, the association between specific MRI assessment features and MVI observed in our study is consistent with findings from previous research. For example, features such as peritumoral enhancement and pseudocapsule presence, which were identified as significant predictors of MVI in our model, have also been reported in other studies to be linked to tumor invasiveness and angiogenesis (21). These features are believed to reflect increased vascular permeability and the formation of a fibrous capsule, both of which are associated with more aggressive tumor behavior.

However, our findings regarding certain radiomics features, such as texture and shape parameters, differ from some previous studies. For instance, while several studies have highlighted the predictive power of first-order statistics such as entropy and skewness in identifying MVI (22, 23), our analysis found that higher-order texture features and wavelet-based features played a more prominent role. This discrepancy may be due to differences in cohort characteristics or the use of a more advanced machine learning model, such as the Transformer architecture, which may be better suited to capture complex, non-linear interactions between radiomic features and MVI (24).

Additionally, our study’s use of multi-center data may explain some differences from single-center studies. The heterogeneity of data across multiple centers can introduce variability that is more reflective of real-world clinical scenarios, potentially leading to different patterns of feature importance. For example, previous single-center studies may not have fully accounted for the influence of different imaging protocols and patient populations, which our model has been trained to manage (17). This makes our model more generalizable, though further prospective validation is necessary to confirm these findings.

While our study offers several strengths, such as the use of multi-center data and advanced modeling techniques, it also has important limitations. Although external validation strengthens the generalizability of our results, the relatively modest sample size, despite being multi-centered, may affect the broader applicability of the findings. Future studies with larger and more diverse cohorts are needed to further validate the model across different populations. Additionally, while our study integrates clinical indicators, MRI assessments, and radiomics, it does not introduce entirely new methodologies or groundbreaking theories. The approach follows established techniques and, as such, does not represent a major theoretical advancement in the field. The combination of these established techniques, however, provides incremental improvements in prediction accuracy, which we believe can still offer significant clinical value. Another limitation is the retrospective design, which may introduce inherent biases. While we have addressed potential bias due to imbalanced data using SMOTE to ensure balanced class distribution during model training, this remains a potential limitation. Future research should adopt prospective designs to mitigate this issue and further enhance the robustness of the model. Furthermore, while MRI assessments are a valuable modality in this study, they may not be universally available in all clinical settings, limiting the general applicability of the model. Expanding the model to incorporate other imaging modalities, such as CT or PET, and integrating novel biomarkers could further enhance its predictive power and applicability. Despite these limitations, our findings underscore the need for integrating advanced computational techniques, such as radiomics and machine learning, into oncological research. The use of multi-center data in this study serves as a foundation for future research, encouraging the exploration of additional data sources and modalities to further improve prediction models for MVI.

Future research should aim to refine the model by incorporating molecular and genetic data, which could provide deeper insights into the biological mechanisms driving MVI. Additionally, clinical trials should focus on validating the model’s performance in real-world clinical settings and across diverse patient populations to ensure its utility in clinical practice.

In conclusion, this multi-center study demonstrates the effectiveness of an integrated approach that combines clinical, MRI assessments, and radiomics features to predict MVI in solitary HCC. The strong performance of the combined model in both internal and external validation sets highlights its potential for improving clinical decision-making and patient outcomes. As radiomics and machine learning techniques continue to evolve, their integration into routine clinical practice holds promise for enhancing the accuracy and utility of cancer prediction models, ultimately leading to better patient management strategies.

## Data Availability

The original contributions presented in the study are included in the article/**Supplementary Material**. Further inquiries can be directed to the corresponding author.
