# Supplementary material for: Predicting microvascular invasion in solitary hepatocellular carcinoma: a multi-center study integrating clinical, MRI assessments, and radiomics indicators
Source: Front Oncol. 2025 Feb 19;15:1511260. doi: 10.3389/fonc.2025.1511260 (PMC11879948; doi:10.3389/fonc.2025.1511260)
Supplement: Supplementary file 1 [file DataSheet1.pdf]

Supplementary Material

1. Supplementary Figures

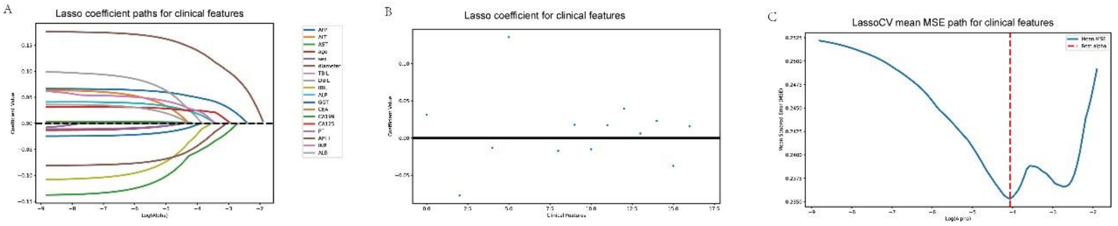

Supplementary Figure 1. Lasso Regression for Clinical Features. (A) Lasso coefficient paths for clinical features. (B) Final Lasso coefficients after selection, showing the most important clinical features for predicting microvascular invasion (MVI). (C) Mean squared error (MSE) path from cross-validation (LassoCV), indicating the optimal point for feature selection. This figure illustrates the Lasso regression process specifically for clinical indicators, identifying the key clinical features that contribute to the prediction of MVI.

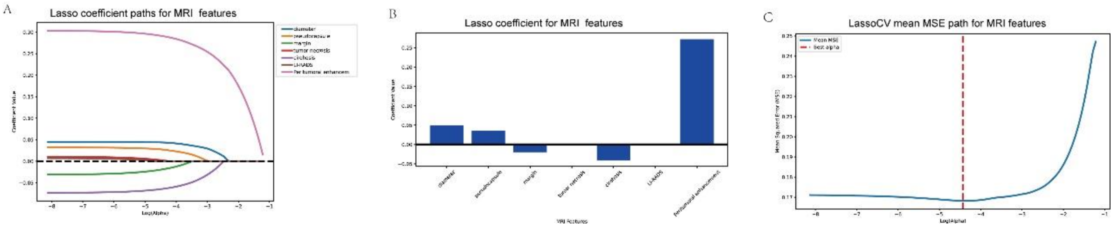

Supplementary Figure 2. Lasso Regression for MRI Features. (A) Lasso coefficient paths for MRI features. (B) Final Lasso coefficients after selection, highlighting the most important MRI features for predicting microvascular invasion (MVI). (C) Mean squared error (MSE) path from cross-validation (LassoCV), showing the optimal feature selection point. This figure demonstrates the Lasso regression process for MRI features, identifying the key MRI characteristics that play a significant role in predicting MVI.

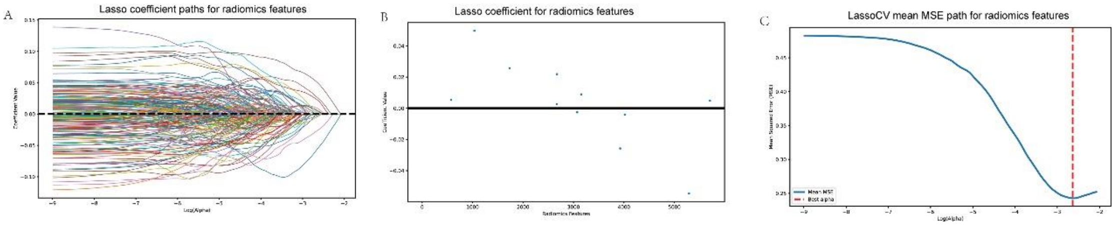

Supplementary Figure 3. Lasso Regression for Radiomics Features. (A) Lasso coefficient paths for radiomics features. (B) Final Lasso coefficients after selection, identifying the most significant radiomics features for predicting microvascular invasion (MVI). (C) Mean squared error (MSE) path from cross-validation (LassoCV), showing the optimal point for feature selection. This figure showcases the Lasso regression process specifically for radiomics features, helping to pinpoint the most critical features for predicting MVI based on radiomic data.

2. Supplementary Table

Supplementary table 1. Basic characteristics in training cohort

	**Total**	**non-MVI**	**MVI**	**P-value**
**Diameter(cm)**	3.3±2.0	2.6±1.6	4.0±2.2	< 0.001
**Age(years)**	57.1±10.7	56.1±10.5	58.0±10.8	0.29
**ALT(U/L)**	34.2±21.2	35.7±25.0	32.7±16.8	0.9
**AST(U/L)**	35.5±25.5	37.5±30.3	33.6±19.9	1
**TBIL (umol/L)**	14.9±7.9	14.9±7.1	14.9±8.6	0.58
**DBIL (umol/L)**	5.5±3.2	5.5±3.0	5.5±3.4	0.92
**IBI (umol/L)**	9.4±5.4	9.5±4.8	9.4±5.9	0.54
**ALP(U/L)**	88.4±33.3	87.3±26.4	89.5±38.9	0.97
**GGT(U/L)**	68.0±114.2	75.0±156.4	61.2±46.3	0.2
**PT(s)**	13.5±1.1	13.4±1.0	13.5±1.3	0.86
**APTT(s)**	35.8±3.1	35.7±3.0	35.8±3.2	0.89
**INR**	1.0±0.1	1.0±0.1	1.0±0.1	0.81
**ALB (g/L)**	42.1±3.9	42.2±3.8	42.0±4.1	0.98
**AFP (ug/L)**	1289.4±6494.5	295.2±823.8	2246.9±8986.5	0.19
**CEA (ng/ml)**	2.8±2.1	2.7±2.1	2.9±2.0	0.22
**CA199 (U/ml)**	20.2±18.0	18.8±18.5	21.6±17.5	0.069
**CA125 (U/ml)**	23.2±91.1	15.9±17.1	30.2±126.5	0.31
**LI-RADS**				
4	16 (10.1%)	10 (12.8%)	6 (7.4%)	0.3
5	143 (89.9%)	68 (87.2%)	75 (92.6%)	
**Sex**				
Female	23 (14.5%)	12 (15.4%)	11 (13.6%)	0.82
Male	136 (85.5%)	66 (84.6%)	70 (86.4%)	
**Tumor necrosis**				
no	151 (95.0%)	73 (93.6%)	78 (96.3%)	0.49
yes	8 (5.0%)	5 (6.4%)	3 (3.7%)	
**Pseudocapsule**				
no	150 (94.3%)	74 (94.9%)	76 (93.8%)	1
yes	9 (5.7%)	4 (5.1%)	5 (6.2%)	
**Cirohosis**				
no	49 (30.8%)	31 (39.7%)	18 (22.2%)	0.025
yes	110 (69.2%)	47 (60.3%)	63 (77.8%)	
**Margin**				
smooth	87 (54.7%)	67 (85.9%)	20 (24.7%)	< 0.001
coarse	72 (45.3%)	11 (14.1%)	61 (75.3%)	
**Peritumoral enhancement**				
no	68 (42.8%)	31 (39.7%)	37 (45.7%)	0.52
yes	91 (57.2%)	47 (60.3%)	44 (54.3%)	
**History of family**				
no	129 (81.1%)	61 (78.2%)	68 (84.0%)	0.42
yes	30 (18.9%)	17 (21.8%)	13 (16.0%)	

ALT: Alanine Aminotransferase, AST: Aspartate Aminotransferase, TBIL: Total Bilirubin, DBIL: Direct Bilirubin, IBIL: Indirect Bilirubin, ALP: Alkaline Phosphatase, GGT: Gamma-Glutamyl Transferase, PT: Prothrombin Time, APTT: Activated Partial Thromboplastin Time, INR: International Normalized Ratio, ALB: Albumin, AFP: Alpha-Fetoprotein, CEA: Carcinoembryonic Antigen, CA199: Carbohydrate Antigen 19-9, CA125: Carbohydrate Antigen 125, LI-RADS: Liver Imaging Reporting and Data System
